# Patient and family involvement in adult critical and intensive care settings: a scoping review

**DOI:** 10.1111/hex.12402

**Published:** 2015-09-07

**Authors:** Michelle Olding, Sarah E. McMillan, Scott Reeves, Madeline H. Schmitt, Kathleen Puntillo, Simon Kitto

**Affiliations:** ^1^ British Columbia Centre for Excellence in HIV/AIDSVancouverBCCanada; ^2^ Collaborative Academic PracticeUniversity Health NetworkTorontoONCanada; ^3^ Centre for Health and Social Care ResearchKingston University and St. George's University of LondonLondonUK; ^4^ School of NursingUniversity of Rochester Medical CentreRochesterNYUSA; ^5^ EmeritaDepartment of Physiological NursingUCSFSan FranciscoCAUSA; ^6^ Department of Innovation in Medical EducationFaculty of MedicineUniversity of OttawaOttawaONCanada

**Keywords:** Critical care, family involvement, family‐centred care, Intensive care units, patient involvement, patient‐centred care, scoping review

## Abstract

**Background:**

Despite international bodies calling for increased patient and family involvement, these concepts remain poorly defined within literature on critical and intensive care settings.

**Objective:**

This scoping review investigates the extent and range of literature on patient and family involvement in critical and intensive care settings. Methodological and empirical gaps are identified, and a future agenda for research into optimizing patient and family involvement is outlined.

**Methods:**

Searches of MEDLINE, CINAHL, Social Work Abstracts and PsycINFO were conducted. English‐language articles published between 2003 and 2014 were retrieved. Articles were included if the studies were undertaken in an intensive care or critical care setting, addressed the topic of patient and family involvement, included a sample of adult critical care patients, their families and/or critical care providers. Two reviewers extracted and charted data and analysed findings using qualitative content analysis.

**Findings:**

A total of 892 articles were screened, 124 were eligible for analysis, including 61 quantitative, 61 qualitative and 2 mixed‐methods studies. There was a significant gap in research on patient involvement in the intensive care unit. The analysis identified five different components of family and patient involvement: (i) presence, (ii) having needs met/being supported, (iii) communication, (iv) decision making and (v) contributing to care.

**Conclusion:**

Three research gaps were identified that require addressing: (i) the scope, extent and nature of patient involvement in intensive care settings; (ii) the broader socio‐cultural processes that shape patient and family involvement; and (iii) the bidirectional implications between patient/family involvement and interprofessional teamwork.

## Background

Critical care research, policy and best practice increasingly recognize that patients admitted to acute care hospitals are members of a wider patient–family network^1^ that functions as a small social system.^2^ The acknowledgement of family members, in this form, marks a departure from the disease‐centric practice of solely focusing on the physiological care of an individual patient within the intensive care unit (ICU).^3^ This new way of thinking is not without contestation as tensions can arise between traditional models of care provision in ICUs and a holistic incorporation of patients, family members and their life worlds into care. Examples of this tension can be illustrated by controversies over whether family members should be allowed to observe cardiopulmonary resuscitation (CPR) of their loved one,^4^ and whether family members should have the opportunity to participate in professional rounds where patient status and treatment plans are discussed.^5^

The movement towards patient involvement is evident in a number of supranational policy statements and directives drafted since the late 1970s^6^, ^7^ and can be situated within a broader rise in health consumerism,^8^ and shift towards patient‐centred models of care.^9^, ^10^, ^11^ The Institute of Medicine's 2001 report *Crossing the Quality Chasm* was a seminal document in acknowledging patient‐centred care as a key component of health‐care quality.^12^ Proponents of patient‐centred care models have since advocated for patient involvement as an intrinsically important health‐care goal that is also instrumental to clinical decision making, quality of care and patient outcomes.^13^, ^14^, ^15^ International bodies such as the World Health Organization have called for variations of a patient and family‐centric model of health‐care delivery,^16^ and the Society of Critical Care Medicine, America's largest non‐profit critical care organization, has developed clinical practice guidelines for the support of family members in the ICU to meet these mandates.^17^

Despite international bodies calling for increased patient and family involvement, this concept remains unclear within the critical care literature. Little consensus exists on what involvement actually means to varying stakeholders,^18^ who at times have different perceptions of manner and degree to which patient and family involvement should take place.^19^ Questions around the nature and extent of patient and family involvement can be fraught with tension due to the environment of the intensive care unit. This setting is characterized by a high level of care provision, close monitoring, and the use of complex medical procedures and equipment in a context where the patient's health status is often severe and unpredictable.^20^ Involvement as a concept is both complex and dynamic and can encompass not only visible activities and interactions between social actors, but also the thoughts feelings, and meanings individuals have towards these activities and interactions.^18^ The conceptual ambiguity surrounding involvement poses problems in terms of facilitating collaborative relationships between patients, families and providers as well as planning, implementing and evaluating initiatives that promote patient and family‐centred care.

Recent literature reviews on family members' involvement in the ICU have primarily focused on family needs^21^, ^22^ and experiences^23^; however, these reviews give little insight into how patient and family involvement is actually being researched across the literature. For instance, we found a lack of research regarding the relationship between patient and family involvement and interprofessional collaboration in intensive care units. Although there is a considerable amount of literature on dyadic communication between family members and health‐care providers,^24^ a very limited number of studies have been conducted on the day‐to‐day involvement of patients and families with multiple health‐care team members.^2^, ^25^, ^26^

The purpose of this scoping review is to map out the extent and range of literature on patient and family involvement in critical and intensive care settings, with attention to key concepts, topics and methodological approaches. More specifically, the scoping review aims to identify empirical and methodological gaps within the existing literature in order to inform an emerging research agenda in patient and family involvement and interprofessional collaboration.

## Methods

Scoping reviews are an exploratory review methodology used to rapidly map the literature on a well‐defined topic, reveal methodological and empirical gaps within a body of research and identify critical areas for investigation.^27^ Scoping reviews are more exploratory and less systematic than systematic reviews, allowing for a broader mapping of varying evidentiary levels of existing research that can inform the development of research questions to guide systematic reviews and empirical studies.^28^ This review was primarily targeted towards reviewing empirical and methodological limitations in order to establish whether there are any gaps in knowledge around patient and family involvement that require the formulation and pursuit of new research questions. We defined a critical care setting to be a hospital unit that provides intensive care medicine to patients with life‐threatening injuries and illnesses. We used Arksey & O'Malley's well‐established framework to undertake our scoping review. This framework consists of five steps: (i) identifying the research questions, (ii) identifying relevant studies, (iii) selecting studies, (iv) charting the data and (v) collating, summarizing and reporting results.^27^

### Identifying the research question

The research questions that guided this review were developed in collaboration with researchers and the advisory board on a larger study examining interprofessional collaboration and patient and family involvement in intensive care settings.^25^ This review investigates the following: What is the extent and range of literature on patient and family involvement in critical and intensive care settings, and what empirical and methodological gaps exist within this literature? In this review, we purposely adopted the term ‘involvement’. We conceived involvement to be a broad term that could encompass other similar concepts such as participation, engagement, inclusion, and empowerment; which is reflective of long‐standing discussions centring on patient and public involvement in health services and research.^29^ To ensure we were comprehensive in our review of the critical care literature, we included variations of these terms in our search strategy. All studies using qualitative, quantitative and mixed‐methods study designs were eligible for inclusion.

### Identifying relevant studies

Studies were selected for this review through searches conducted on OVID MEDLINE, CINAHL, PyschINFO and Social Work abstracts. These databases were used to reach a broad range of English‐language literature published in the last decade (2003–2014) within peer‐reviewed health and social science journals. This publication range was selected to provide insight into the expansion of literature and interest in this topic during this particular period. Two reviewers developed the search strategies (see Table 1) in consultation with a health information scientist. In addition to these searches, the reviewers examined the reference list of an existing literature review on patient and family involvement to identify eligible articles that may have been missed by the searches.^22^ Members of an expert advisory group were consulted to identify any remaining eligible articles not picked up by the search or reference list search.

**Table 1:** Search strategies

Database	Search term syntax
MEDLINE	(“Critical Care” [MESH terms] OR Intensive Care Units [MESH Terms]) AND (“Patients” [MESH Terms] or “Family” [MESH Terms] or “Caregivers” [MESH Terms]) AND (involvement or engagement or collaboration or experience or empowerment or interactions or perceptions or presence or needs or visitation or advocacy).mp. AND LIMIT TO (english language and humans and yr=“2000 –Current” and “all adult (19 plus years)”)) AND NOT (“Intensive Care Units, Pediatric [MESH Terms] OR “Intensive Care Units, Neonatal” [MESH Terms])
CINAHL	((MH ‘Intensive care units’ OR MH ‘Critical Care’) AND (MH Patients OR MH Physicians, Family OR MH Patient‐Family Relations OR MH Family OR MH Extended Family OR MH Family Relations) AND (TX involvement OR TX engagement OR TX collaboration OR TX experience OR TX empowerment OR TX interactions OR TX perceptions OR TX presence OR TX needs OR TX visitation OR TX advocacy)
PsychINFO	(critical care OR intensive care unit) AND (patient OR family OR caregiver) AND (involvement OR engagement OR collaboration OR experience OR empowerment OR interactions OR perceptions OR presence OR needs OR visitation OR advocacy)).mp. AND LIMIT TO (full text and peer reviewed journal AND human AND english language AND abstracts AND ‘300 adulthood <age 18 yrs and older>’ AND ‘0110 peer‐reviewed journal’) AND NOT ((pediatric or paediatric.mp. or neonatal.mp.) OR exp Neonatal Intensive Care/OR exp Pediatrics/)
Social Work Abstracts	((critical care or intensive care unit) AND (patient or family or caregiver) and (involvement or engagement or collaboration or experience or empowerment or interactions or perceptions or presence or needs or visitation or advocacy)).mp.

### Selecting studies

In the first stage of selection, two reviewers read through article abstracts to eliminate duplicates and exclude ineligible articles. Studies were included if they were set in an intensive care or critical care setting, addressed the topic of patient and family involvement, and included a sample of adult critical care patients, their families and/or critical care providers. Articles were excluded from the study if they were commentaries or editorials; prevalence studies of mental health conditions in the ICU; paediatric studies; studies that had no reference to relationships between providers, families, patients; or validation studies. As study abstracts often lacked critical information about the study methodology and setting, the reviewers assessed the full text of remaining articles using the same study criteria.

The two reviewers ensured consistency in inclusion and exclusion decisions by independently applying the criteria to an initial sample of approximately 20 manuscripts and subsequently comparing and discussing any differences in their decisions about inclusion/exclusion. This initial piloting of criteria helped clarify decision making around inclusion/exclusion and ensured that criteria were applied consistently. The pilot coding revealed that there was a strong intercoder agreement between the two reviewers. Following the pilot coding, the remaining articles were divided between the two reviewers to expedite the process. When a reviewer was uncertain about whether a study met inclusion criteria, he/she discussed the study with the second reviewer to achieve consensus.

### Charting the data

The reviewers charted articles by extracting relevant information on study aim, setting, design/method and population. In the majority of cases, the reviewers were able to identify the research design from the abstract; in cases where methodology was descriptive or vague, the reviewers interpreted the research designs according to the description within the methods and results section. In the latter cases, both reviewers examined the articles to ensure consensus. The articles were coded to chart the type of terminology used to describe patient and family involvement in the article.

### Collating, summarizing and reporting results

A qualitative content analysis was adopted for summarizing and synthesizing the characteristics of studies included within this scoping review. We produced numerical summaries to map the overall number of studies, settings and methodologies. A conventional content analysis^30^ was then used to inductively identify patterns in the ways patient and family involvement was described within the articles included in this review. Conventional content analysis entails developing codes inductively through immersion with the text, deriving codes from the data itself rather than coding with pre‐conceived categories.^30^ This dual coding process allowed us to comment on general methodological trends across the literature, addressing regularities and gaps, as well as to thematically describe components of both patient and family involvement.^31^

## Results

### Study selection

**Figure 1:**
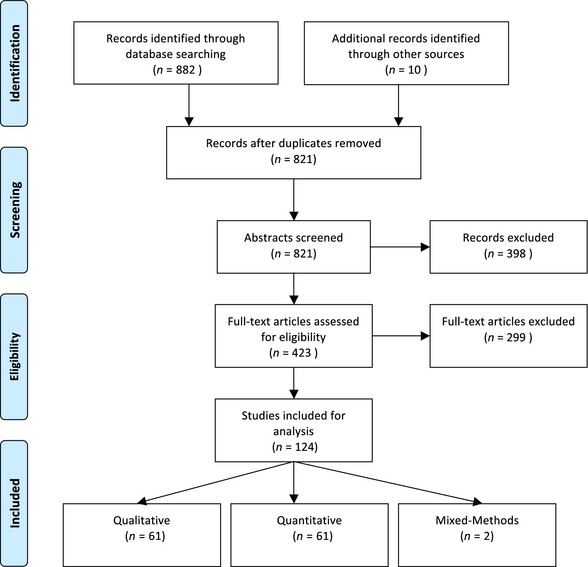
Flow diagram of study methodology. This flow diagram illustrates our study selection process, which culminated in a total count of 124 included studies. [Colour figure can be viewed at wileyonlinelibrary.com]

The review searches initially yielded a total of 882 articles. After removing 71 duplicates, the two reviewers excluded 398 ineligible articles through the abstract review and an additional 299 through the full‐text assessment. The review of reference lists retrieved 6 articles and a consultation with experts on the advisory group retrieved another 4, bringing the total count of included studies to 124 (See Fig. 1).

### Study characteristics

Of the 124 studies included, 61 are quantitative, 61 are qualitative and 2 are mixed‐methods studies. Findings on study characteristics are reported in Table 2. The most common research design employed to study patient and family involvement within the quantitative studies is the cross‐sectional survey, which accounts for 73.8% of all quantitative studies (*n* = 45). The quantitative literature also includes 10 observational studies, 4 pre–post studies, 1 randomized controlled trial and 1 non‐randomized controlled trial. Amongst the 61 qualitative studies, most are described as interview‐based exploratory qualitative designs (*n* = 21). The qualitative literature also includes 17 interview‐based phenomenological studies, 9 ethnographic studies, 2 grounded theory studies and 2 action research studies.

**Table 2:** Summary of study characteristics

Variable	Total – *N* (%)
Total – *N*	124
Setting of study – Continent	
Africa	0 (0.0)
Asia	11 (8.9)
Australasia (Australia and NZ)	7 (5.6)
Europe	45 (36.3)
North America	59 (47.6)
South America	2 (1.6)
Journal type	
Nursing	79 (63.7)
Anesthesiology	2 (1.6)
Critical care medicine	32 (25.8)
Qualitative health research	1 (0.8)
Social work	1 (0.8)
Psychology	0 (0.0)
Other	9 (7.5)
Study design	
Quantitative	61 (49.2)
Randomized controlled trial	1 (0.8)
Non‐randomized control trial	1 (0.8)
Pre–post	4 (3.2)
Observational	10 (8.1)
Cross‐sectional	45 (36.3)
Other	0 (0.0)
Mixed methods	2 (1.6)
Qualitative	61 (49.2)
Action Research	2 (1.6)
Case study	0 (0.0)
Ethnography	9 (7.3)
Grounded theory	12 (9.7)
Phenomenology	17 (13.7)
Qualitative (other/not specified)	21 (16.9)
Analysis	
Statistical	63 (50.8)
Content analysis	17 (13.7)
Thematic analysis	17 (13.7)
Discourse analysis	1 (0.8)
Grounded theory/constant comparative method	14 (11.3)
Phenomenological/hermeneutical analysis	8 (6.5)
Other/not specified	4 (3.2)

The papers are overwhelmingly published in journals targeted at nursing audiences (*n* = 79), with most of the remaining studies published in critical care medicine journals (*n* = 32). In terms of geographical distribution, the United States is the leading site for research (*n* = 48), followed by Sweden (*n* = 22), Canada (*n* = 7), Australia (*n* = 6) and Norway (*n* = 6). The participants for these studies are most commonly family members of critically ill patients (*n* = 41) or nurses (*n* = 35) (Table 3).

**Table 3:** Study participants/professional groups

Participants/professional groups	Total – *N* (%)
Total – *N*	124
Nurses	35 (28.2)
Nurses and family members	6 (4.8)
Nurses, family and patient	1 (0.8)
Nurses and physicians	2 (1.6)
Nurses and physicians and family members	3 (2.4)
Physicians and family members	4 (3.2)
Physicians and patients	2 (1.6)
Interprofessional staff	5 (4.0)
Interprofessional staff and family members	4 (3.2)
Interprofessional staff, family members and patients	3 (2.4)
Family members	41(33.1)
Family members and patients	5 (4.0)
Patients	13 (10.5)

### Patient involvement

In regard to patient involvement, the two key components of patient involvement investigated within the literature are as follows: (i) patient experience and (ii) patient participation. The variety and volume of research conducted on family involvement far surpasses that on patient involvement. Notably, ‘patient involvement’ is a concept that has not been significantly explored in critical care research, with only six qualitative articles retrieved on the topic. Three of these studies focus broadly on ‘patient experience’, through interviews with patients, and only peripherally discuss any aspect of patient involvement in communication or decision making.^32^, ^33^, ^34^ Two of these studies note that mechanically ventilated patients who were able to participate in some form in their care, expressed feeling less like an object, increased in dependence and positivity towards their recovery, and also felt that time passed more quickly.^33^, ^34^ However, Karlsson and colleagues also offer up critical questions around the extent to which patients may be able to participate in decision making about their care in the ICU, arguing there may be a ‘fine line between a challenge and too much pressure on the vulnerable patient’.^33^

Two other studies employ interviews and focus groups with nurses to explore their perspectives on communication with patients,^35^ and patient participation in decision making.^36^ In Trovo de Arujo and Da Silvia's study exploring 10 Sao Paulo nurses’ perceptions of communication with patients, the authors find that while nurses’ valued communication with patients as a therapeutic resource in palliative care, they felt ill prepared to communicate with dying patients. They identify uncertainty around patient awareness as a common obstacle to communication with patients.^35^ Kvangarsnes and colleagues’ study similarly find that nurses considered patient participation in decision making to be especially challenging during life or death situations such as chronic obstructive pulmonary disease exacerbation and that they felt patients had low levels of power or involvement in their treatment at this stage.^36^ The sixth and last study uses ethnographic approaches to identify the nature and scope of patient involvement in an American ICU and concluded with recommendations to empirically explore several aspects of ICU patients’ involvement in decision making.^37^

The patient involvement studies reviewed tend to focus on the ability of patients to communicate with providers and family members. In investigating the experience of ICU patients, however, the findings from these studies shed light on contextual factors that limited patient involvement in intensive care units. These factors include technologically intensive ICU environments, clinical objectification of patients, voicelessness and breathlessness caused by intubation, and assumptions around cognitive ability and illness severity.^32^, ^33^, ^34^, ^35^, ^36^, ^37^

### Family involvement

**Figure 2:**
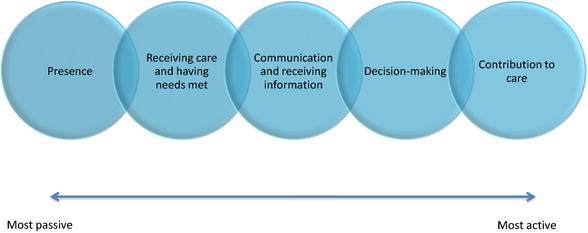
This diagram depicts the components of family involvement investigated within the empirical literature on intensive care units. These categories of involvement are not mutually exclusive, but rather represent subsequent and progressive components of involvement along a continuum from relatively passive to active forms. [Colour figure can be viewed at wileyonlinelibrary.com]

We identified five main components of family involvement that have been investigated within the studies on intensive care units. We propose that these categories of involvement are not mutually exclusive, but rather represent aspects of involvement that range along a continuum from relatively passive to active involvement. (Fig. 2) Dreyer & Nortvedt describe four increasing stages of involvement that family carers of medically sedated patients in the ICU move through from admittance to discharge.^38^ Although we also describe involvement in terms of a range, we do not mean to infer these components are taken up in a strict linear trajectory. Our analysis is meant to take into account the inter‐related, dynamic and recursive nature of patient and family involvement under investigation. The five components of family involvement, discussed below in order of prominence within our scoping review, are as follows: (i) Involvement as presence; (ii) Involvement as receiving care and having needs met; (iii) Involvement as communicating and receiving information; (iv) Involvement as decision making; and (v) Involvement as contributing to care.

#### Presence and visitation (*n* = 40)

‘Family presence’ is a component of family involvement that has been extensively researched within the critical care literature, accounting for nearly one‐third of all included studies. The term ‘presence’ connotes a relatively passive role for families as visitors to the ICU,^39^, ^40^, ^41^, ^42^, ^43^, ^44^, ^45^, ^46^, ^47^, ^48^ attendants at the patient's bedside^49^ or witnesses to invasive procedures.^4^, ^50^, ^51^, ^52^, ^53^, ^54^, ^55^, ^56^, ^57^, ^58^, ^59^, ^60^, ^61^, ^62^, ^63^ Amongst the 23 quantitative studies, the majority (*n* = 16) measure provider perspectives, attitudes and preferences towards family presence during resuscitation.^4^, ^19^, ^50^, ^51^, ^52^, ^53^, ^54^, ^55^, ^56^, ^57^, ^58^, ^59^, ^60^, ^63^, ^64^ These studies predominantly examine the perspective of nurses,^4^, ^19^, ^50^, ^51^, ^52^, ^53^, ^54^, ^59^, ^60^, ^61^, ^63^ although five compare the perspectives of nurses and physicians.^19^, ^52^, ^55^, ^60^, ^64^ The quantitative literature examining family member or patient perspectives on presence in the ICU is considerably slimmer, with only 2 articles retrieved.^57^, ^61^ The remaining quantitative studies all use survey methods and include one study assessing nurse attitudes towards visitation,^44^ one study describing attitudes and perceptions of multidisciplinary staff towards family presence during bedside rounds,^49^ one study examining the association between family presence and environmental factors at the time of a patient's death,^65^ and three studies that examine the relationship between family visitation and patient outcomes^39^ or family well‐being.^40^, ^43^ Amongst the sixteen qualitative studies on family presence, all use in‐depth interviews to explore the content and meaning of family presence and visitation in the ICU from the perspective of family members,^46^, ^62^, ^66^, ^67^ patients^46^, ^47^, ^68^, ^69^, ^70^ or providers.^69^, ^71^, ^72^, ^73^, ^74^, ^75^

#### Receiving care and having needs met (*n* = 33)

Another research topic that features prominently in the included literature is the identification of family needs in the ICU. Family members of critically ill patients are routinely represented within the literature as recipients of care with distinct psychological, social and physical needs.^22^, ^76^ As such, critical care researchers describe the identification and satisfaction of family needs as a pre‐requisite for effective partnerships between families and providers.^20^, ^21^, ^23^ Within the family needs literature, 1 mixed‐methods and 19 quantitative studies adopt cross‐sectional designs to measure the importance of different family needs.^20^, ^77^, ^78^, ^79^, ^80^, ^81^, ^82^, ^83^, ^84^, ^85^, ^86^, ^87^, ^88^, ^89^, ^90^, ^91^, ^92^, ^93^, ^94^, ^95^, ^96^ All except one of these studies use the Critical Care Family Needs Inventory (CCFNI), a 45 item self‐report questionnaire that assesses family needs within five dimensions: support, comfort, information, proximity and assurance.^97^ While the CCFNI has been primarily used in English‐speaking countries, adapted versions of the CCFNI have also been used to identify family needs in Israel,^89^ Greece,^83^ Hong Kong,^57^, ^85^ Brazil^84^ and Jordan.^92^ In total, thirteen studies use qualitative methods to explore family member needs, five of which investigate nurses’ perspectives.^98^, ^99^, ^100^, ^101^, ^102^, ^103^, ^104^, ^105^, ^106^, ^107^, ^108^, ^109^, ^110^

#### Communication and receiving information (*n* = 17)

The third largest research body is the study of communication between patients, providers and families in the ICU. The communication and information literature relates closely to family needs literature in that many of these studies seek to explore how family members perceived and used informational support from health‐care providers.^24^, ^94^, ^111^, ^112^, ^113^, ^114^ The 9 quantitative studies primarily explore how timing, type, quantity or consistency of communication between providers and family members related to family member's satisfaction,^5^, ^115^ prognostic estimation,^111^, ^114^ decision making^113^, ^116^ and the quality of care.^117^, ^118^, ^119^ The 8 qualitative studies more broadly identify, describe and interpret patterns of communication and interaction in the ICU.^24^, ^104^, ^120^, ^121^, ^122^, ^123^, ^124^, ^125^

#### Decision making (*n* = 17)

During the course of a patient's stay in the ICU, family members must often assume responsibility over health‐related decision making, including choices about diagnostics, treatment and therapeutic care. Family member involvement in decision making was the subject of 7 quantitative^112^, ^116^, ^126^, ^127^, ^128^, ^129^, ^130^ and 10 qualitative studies.^131^, ^132^, ^133^, ^134^, ^135^, ^136^ Amongst the qualitative studies, five use in‐depth interviews to explore how surrogate‐decision‐makers participate in decision making around life support.^131^, ^132^, ^133^, ^134^, ^135^ Three other articles emerge from an ethnographic study in which investigators study end‐of‐life decision making (EOLDM) in four adult medical and surgical ICUs within one hospital. These studies explore differences in unit cultures surrounding EOLDM,^26^ the implications of rotating ‘attending physician’ roles on family involvement^137^ and the informal roles family members enacted during the process of EOLDM.^2^ In another qualitative study, investigators interview nurses on their perceived role in family–team conflicts related to treatment plans.^138^ The remaining qualitative study uses in‐depth interviews with family members to identify personal, social and care‐related factors influencing surrogate‐decision‐makers' stress.^136^

Amongst the quantitative studies, two are longitudinal studies in which investigators examine factors associated with surrogate‐decision‐makers’ satisfaction.^112^, ^127^ The remaining quantitative studies include a non‐randomized RCT evaluating an intervention to mitigate decisional conflict,^128^ a prospective study to identify predictors of team–family conflict around treatment plans,^139^ a chart audit to examine family involvement in end‐of‐life decision making,^130^ a cross‐sectional survey to assess family members’ opinions about participating in medical decision making,^126^ and a cross‐sectional survey that examines the frequency with which family members were informed of end‐of‐life decisions (EOLD).^65^

#### Contribution to care (*n* = 12)

The fifth and least researched component of family involvement in the critical care research is family member contribution to patient care. In eleven qualitative studies and one quantitative study, researchers seek to identify and explore the contributions that family members made to patient care.^38^, ^140^, ^141^, ^142^, ^143^, ^144^, ^145^, ^146^, ^147^, ^148^, ^149^, ^150^ These studies explore tangible contributions to care, such as bathing, massaging and cleaning,^145^, ^146^ as well as more intangible contributions such as social and moral support.^141^, ^144^ In most of the qualitative studies, researchers interview either family members themselves^38^, ^140^, ^141^, ^144^, ^145^, ^150^ or critical care nurses for their perspectives on these contributions.^142^, ^143^, ^148^ The remaining qualitative studies focuses on the experience of nurse–family members^149^ and patients themselves.^147^ In the one quantitative study, researchers analyse the relationship between family members’ contributions to care and their perceptions of provider respect, collaboration and support.^151^

## Discussion

### Knowledge gaps pertaining to family involvement

The widespread shift towards patient and family‐centred care has been characterized by Garrouste‐Orgeas and colleagues as, ‘a global philosophical approach in which families are both recipients of care aimed at optimizing their well‐being and active participants in care provided to the patient’.^40^ However, findings from this scoping review indicate that considerably more research has examined the former aspect (*families as recipients of care*) than the latter (*families as active participants in care*). Where family involvement has been studied, the gaze tends to be oriented towards relatively passive forms of involvement, such as family presence during resuscitation. One implication of this trend is that the family involvement literature often views family members as vulnerable subjects that must be brought into the fold of care (i.e. as patients) or as resources for improving patient outcomes, but very rarely as individuals to be partnered with by health‐care professionals in the care of the patient. As such, the family involvement literature may not be interacting effectively with the hidden care work that family members do in critical care settings,^141^ and the implications of these contributions on patient experience, safety and quality of care. The literature is also missing a critical examination of the barriers and facilitators to partnerships between patients, families and providers in intensive care settings.

### Knowledge gaps pertaining to patient involvement

This scoping review identified a distinct lack of research on the nature and extent of patient participation and involvement in their own treatment and care. Although patient participation has become a pillar of health services research and practice,^18^ the topic is notably absent within the critical care literature. This gap may be partly attributed to dominant conceptions of patient involvement as oral communication and decision‐making capabilities, which do not often match with what is possible for critically ill patients experiencing severe illness, sedation, delirium or blocked airways from intubation.^36^, ^69^, ^152^, ^153^, ^154^

The nature of patient participation in the ICU is particular to the ICU setting and may be less obvious to researchers and health‐care providers than it would be in other health‐care settings. The appointment of a surrogate‐decision‐maker and/or an advanced directive (written treatment plan) are other possible ways in which patients may participate in decision making.^155^ However, patient participation may also take the form of non‐verbal participation, expressed through body language, or even behaviours and actions that are typically viewed by providers as disruptive, such as attempts to remove endotracheal tubes or dialysis catheters.^37^ As another example, some participants in Karlsson and colleagues’ study of conscious mechanically ventilated patients described participating in mental training strategies to become more aware of their surroundings and regain a sense of control.^33^ Given the current scarcity of empirical data, there is a need for more exploratory research into the nature and extent of patient participation in the ICU.

### Knowledge gaps pertaining to socio‐cultural factors shaping involvement

Another limitation of the current literature is the disproportionate focus on provider‐family relationships and provider perceptions as factors affecting patient and family involvement. In particular, the relationship between family members and nurses has received considerable attention, a trend that is common in the broader literature on patient participation^152^ and interprofessional care in intensive care settings.^156^ Although relationships between nurses and family members are significant, we note that the wider processual, organizational and contextual factors that shape the conditions for family involvement are largely under‐researched.

One such factor that likely shapes patient and family involvement, but which has not been investigated extensively, is the built environment of the ICU. Some studies have explored the effects of sound environment on patient experience,^157^ as well as patient and family preference towards hospital design in the ICU.^158^ However, attention to how environments may facilitate or inhibit the involvement of family members in the ICU has not been rigorously investigated in the literature. As an exception to this trend, some Swedish researchers have investigated the question of patient and family experience within the ICU environment.^32^, ^65^ Almerud and colleagues’ study on patient experience in the ICU highlights how the technologically intensive landscapes of ICUs themselves, populated with complex medical equipment, can make ICU environments difficult to understand and navigate for patients and families.^32^ Fridh and colleagues found in their interview‐based study that nurses played an instrumental role in ‘piloting’ family members through the often unfamiliar technology‐intense environment.^65^ While these studies offer useful insights into patient and family interaction with ICU environments, more research is needed to understand how the ICU environmental factors described above directly or indirectly shape the possibility of optimal family and patient involvement in patient care. Patient and family involvement might be studied in the future by observing how health‐care professionals, patients and families conceptualize and interact with the spatial layout of the ICU, as well as the furniture, equipment and other physical artefacts within the space.^159^ By extension, research examining how space within ICUs shapes interactions between health‐care professionals, families and patients in (sub)optimal ways is needed.

The literature on patient and family involvement could also be strengthened by attention to broader contextual factors shaping the setting under investigation. With the exception of a few studies examining cultural preferences of patients^19^, ^107^, ^108^, ^148^ and 1 ethnographic study examining unit cultures and EOLDM processes,^26^ the critical care literature lacks sufficient attention to the ways in which gender, ethnicity, age and socio‐economic status may influence practices and preferences around patient or family involvement. There is some recognition in the literature that cultural differences between the patient and ICU team may lead to misunderstandings or conflict around patient care.^107^, ^108^ However, most investigators stop short of considering the ways in which health organizations’ expectations and practices around family involvement may reflect gendered, ethno‐cultural, and/or class‐based assumptions particular to that setting. As one notable exception, Baggs and colleagues observed different patterns in the timing and nature of EOLDM between medical and surgical ICU, which they link to meaningful differences in unit‐based culture, including informal rules around DNRs, the meaning and uses of technological interventions, physician roles and relationships with families, and processes such as unit rounds.^26^

Just as unit culture shapes the possibilities of patient and family participation, patients and family members bring their own diverse set of experiences, expectations and beliefs about what participation should entail. There is evidence from other health‐care settings to support that patients and their families perceive and conceptualize participation or involvement differently depending on their social position, cultural expectations and previous experiences with health‐care consultation.^2^, ^160^, ^161^, ^162^, ^163^, ^164^ Quinn and colleagues identify 8 different informal roles that family members may enact when responding to the challenge of EOLDM, placing these within situational demands and the personal characteristics of diverse family systems.^2^ However, this kind of consideration of the personal characteristics and experiences of families or patients was often absent in the literature. As Protheroe and colleagues’ contend, ‘current definitions [of participation] fail to refer to equity in the ability and capacity to participate and thus ignore the impact of external contexts, social status and marginalization on the participation’.^160^ Differences in ability to participate likely go beyond issues of health literacy and may reflect deep‐set differences in role expectations within health‐care settings.^160^ A better understanding of these differences, and their social underpinnings, may help inform effective approaches to address disparities in participation.

### Knowledge gaps pertaining to interprofessional workflows and dynamics

A significant gap within the literature is consideration of the ways in which interprofessional dynamics shape opportunities for family–patient involvement and in turn, the implications of patient and family involvement on interprofessional teamwork. As Table 2 and 3 show, nurses have been at the forefront of conducting research on patient and family involvement in the ICU, as well as the primary subjects of study. This trend resonates with the widespread role expectation that nurses play a leading role in facilitating patient and family involvement.^146^, ^165^, ^166^ However, the ability of nurses to facilitate patient and family involvement is complicated by a critical care setting that sits within a broader health‐care system context where the medical profession maintains authority over decision making and allocating labour.^167^, ^168^, ^169^, ^170^ As such, it would seem unlikely that nurses alone have power to create conditions and teamwork dynamics conducive to patient and family involvement. Kvangarsnes and colleagues, for example, found in their research that nurses’ ability to respond to patient and family preferences during critical situations was constrained when no physicians were present to authorize decisions.^36^ Baggs and colleagues describe nurses’ efforts to work around attending physicians who lacked an open attitude and behaviour towards the end‐of‐life decision making with families.^137^ This issue of medical dominance again underscores the importance of understanding local professional and socio‐cultural practices within interprofessional teams and how they may shape the possibilities for patient and family involvement. A future avenue for research, as such, would be to explore differences in providers’ conceptualizations of family involvement, and how particular aspects of involvement affect interprofessional team dynamics.

### The need for methodological triangulation and ethnographic methods

As a final observation, there were very few studies in the included literature that triangulated methodologically, with most studies using either quantitative surveys or qualitative interviews as their sole data collection method. This methodological gap is particularly pronounced in the family needs literature, where investigators relied almost exclusively on the CCFNI survey as a data collection method. The dominance of the CCFNI as a tool to understand family needs has led some researchers to conclude that its five dimensions (support, comfort, information, proximity and assurance) represent a ‘universal and predictable set of needs’ experienced by families in ICUs.^90^ This reliance on CCFNI to assess family needs is problematic given that neither the development nor subsequent validations of CCFNI entailed consultations with family members.^21^ More broadly, there are aspects of ICU experience that are not well understood through quantitative tools. Surveys alone cannot tell us why different aspects of family needs are rated the way they are, nor illuminate the personal experiences and contextual factors that shape these needs.

The lack of data triangulation was also a limiting feature within the qualitative literature, given the common use of provider interviews alone to investigate issues related to family presence, involvement and visitation. Although interviews offer a valuable way to access insider accounts on events within the ICU, there are often meaningful differences between what people *say* happens and what actually happens in practice.^171^, ^172^ Ethnographic approaches, which entail sustained observations and immersions within social settings, can provide additional rigour and nuance to survey and interview‐based approaches by illuminating the social, cultural and professional processes that shape the possibilities for patient and family involvement within particular contexts.^2^, ^26^, ^137^, ^173^, ^174^, ^175^ Ethnographic approaches may be particularly instrumental in studying patient involvement, which is often difficult to investigate through survey and interview methods. A notable example from the literature is Happ and colleagues ‘micro‐ethnography’ of patient involvement in health‐related decisions during prolonged critical illness. Using participant observation, clinical record review, interviews and event analysis, the researchers identified age, gender and health‐related differences in patient involvement. In addition, insights into the extent of how and when patient involvement in decisions was initiated were also revealed. There is a need to build on this kind of research within diverse intensive care settings in order to develop context‐sensitive understandings of patient and family involvement.^156^

### Limitations

There are three key limitations to this review. First, only English‐language articles were considered for inclusion in the study. As such, this review misses potentially relevant articles written in other languages and primarily covers research conducted in North America. Secondly, our review did not target studies on advance directives as an expression of patient involvement, which may partly explain the very small number of articles we retrieved on patient involvement in the ICU. Finally, because the reviewers limited their searches to academic research articles published in the last decade, the scoping review cannot speak to how involvement has been conceptualized within grey literature such as media‐sources, commentaries, policy documents and patient and family education materials. This restriction on grey literature was necessary to limit the volume of articles reviewed and maintain a focus on critical care research. It would be constructive to further investigate the grey literature, given its potential influence on critical care research priorities and clinical practice.

## Conclusion

Through this scoping review, we set out to map out the extent and range of research on patient and family involvement investigated in ICUs. This scoping review identified five main components of family involvement that have been investigated in critical care research: (i) presence and visitation, (ii) having needs met or being supported, (iii) communication/receiving information, (iv) decision making and (v) contribution to care. A key finding to emerge from this review is that patient involvement has not, in fact, received much attention within critical care literature. Where patient involvement has been explored, the focus has been primarily been on communication with families and providers around a limited set of medical decisions. While the movement for family‐centred care calls for families to be incorporated as partners in care,^176^ there is remarkably little research that investigates family member's contributions to care in intensive and critical care settings, including its scope and implications on patient care.

In light of the research gaps identified by this review, we suggest that a future research agenda should focus on the following: (i) the scope, extent and nature of patient involvement in intensive care settings; (ii) the broader socio‐cultural processes that shape patient and family involvement, including processual, organizational and contextual factors; and (iii) the relationship between patient and family involvement and interprofessional teamwork processes. In terms of methodology, future research could be strengthened through incorporation of ethnographic approaches that produce in‐depth, context‐specific accounts of patient and family involvement. We argue that this research agenda will at least move the critical care research literature towards evidence that can inform the creation of context‐sensitive and sustainable interventions to improve the involvement of families and patients in the treatment and care of ICU patients.

## Sources of funding

This study was funded by The Betty and Gordon Moore Foundation, which took no part in the conception, design, conduct, interpretation or preparation of this manuscript. The Betty and Gordon Moore Foundation is located at 1161 Page Mill Road, Palo Alto, CA 94304.

## Conflict of interests

The authors have no conflicts of interest to declare.
